# Cruciate Paralysis Following a Displaced Type II Odontoid Fracture: A Case Report

**DOI:** 10.7759/cureus.25181

**Published:** 2022-05-21

**Authors:** Konstantinos Zygogiannis, Jim Dimitris Georgoulis, Spyros I Antonopoulos, Georgios Gourtzelidis, Ioannis Chatzikomninos

**Affiliations:** 1 Trauma and Orthopaedics, Laiko General Hospital of Athens, Athens, GRC; 2 First Department of Orthopedics, Attikon University General Hospital, National and Kapodistrian University of Athens School of Medicine, Athens, GRC; 3 Orthopaedics, KAT Attica General Hospital, Athens, GRC; 4 Spine and Scoliosis, KAT Attica General Hospital, Athens, GRC

**Keywords:** spine cord, cervical spine fracture, case report, cruciate paralysis, odontoid fracture

## Abstract

A 54-year-old male was admitted to our emergency department by air transport after being hit as a pedestrian by a motorcycle. He presented with impaired motor function in the upper extremities bilaterally while sensation was spared. He presented no motor or sensory impairment of the lower extremities. A computed tomography scan revealed a displaced type II odontoid fracture. Treatment consisted of open reduction and internal fixation of the odontoid with a single screw. The patient’s functional outcome was excellent during the two-month follow-up.

Cruciate paralysis is a relatively rare although well-defined neurological condition which results from injury at pyramid decussation. In this case, the presence of a posterior bony spike of the fractured dens was responsible for the development of cruciate paralysis. Early diagnosis and adequate treatment can have successful results.

## Introduction

First described by Bell in 1970 [1,2], cruciate paralysis is a rare clinical entity induced by injury at the level of cervicomedullary junction (CMJ). The clinical image could be considered paradoxical because often patients present with significant muscle weakness in the upper extremities while sparing the lower extremities [3,4]. As the level of injury is above C4, symptoms may include respiratory failure, cranial nerve deficits, or even a comatose state. The leading speculation derives from the fact that the motor tract fibers of the upper extremities are situated more superior and ventral related to those of the lower extremities, allowing them to act independently [3,4]. Consequently, any injury from the CMJ to the C2, in this case, an odontoid type II fracture with a bony spike, can affect only the upper limbs resulting in this clinical image.

## Case presentation

**Figure 1:**
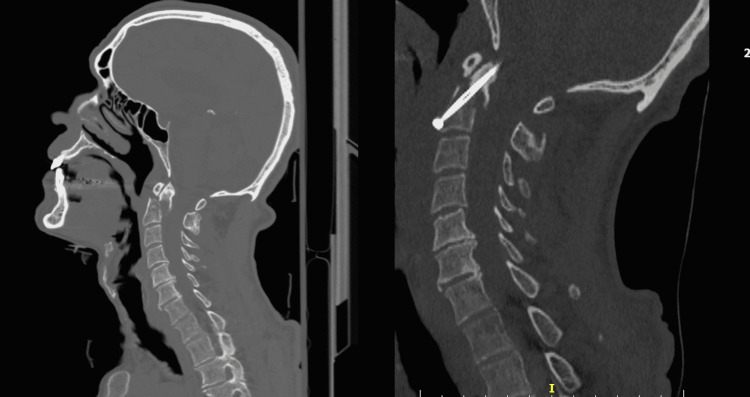
Pre and postoperative computed tomography images of the cervical spine. Left image: posteriorly dislocated type II odontoid fracture with significant step off and evident bony spike. Right image: postoperative single screw fixation with satisfactory reduction.

**Figure 2:**
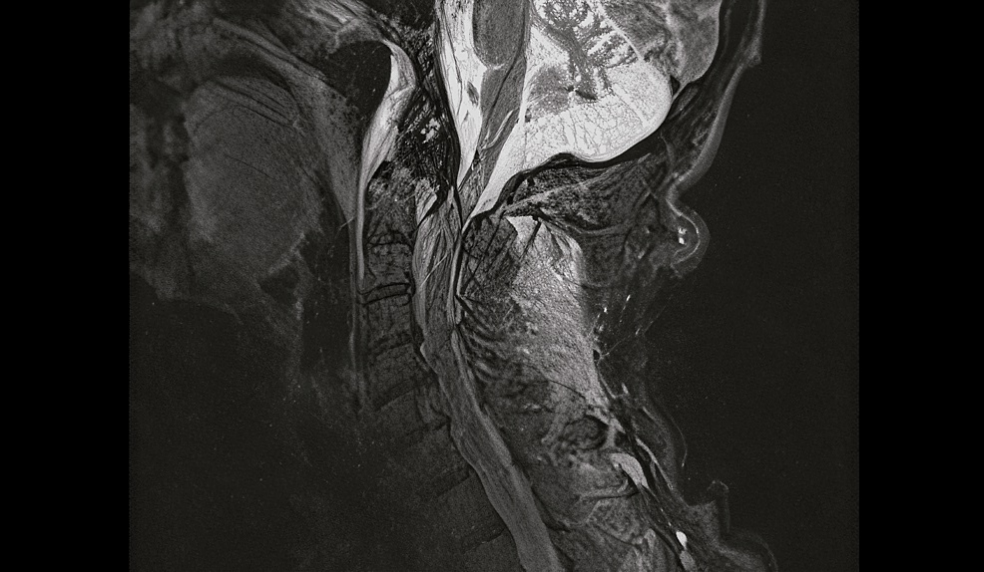
Sagittal T2-weighted magnetic resonance imaging of the cervical spine. Type II odontoid fracture with posterior dislocation causing cord compression.

A 54-year-old Caucasian male was admitted to our Trauma Hospital via air transport after being hit as a pedestrian by a motorcycle. Air transport was ordered as the patient was initially evaluated by local doctors as having incomplete tetraplegia. He presented to us with severe pain in the neck and shoulder region. Clinical examination revealed impaired motor function in the upper extremities bilaterally. There was no sensory deficit and no impairment of the lower extremities. The diagnostic workup that included plain radiographs, computed tomography (Figure 1), and magnetic resonance imaging (Figure 2) revealed a posteriorly displaced type II odontoid fracture (Grauer IIB) with spinal cord compression and fracture of the spinous processes of A6-A7.

Provisional reduction and stabilization with a Halo orthosis were achieved. As the patient was noncompliant to keep the Halo orthosis on the fifth day and no neurological progress was evident, we decided to proceed with open reduction and internal fixation of the odontoid fracture by a single screw on the fifth day of admission. The patient showed some signs of motor improvement on the third postoperative day. At the two-month follow-up, neurological recovery was evident.

## Discussion

Cruciate paralysis is defined as a rare clinical entity that can easily be misdiagnosed as it can simulate acute cervical central cord injury syndrome [3]. Acute central cord syndrome usually occurs by hyperextension of the cervical spine in the presence of spinal canal stenosis. Paradoxical paralysis only of the upper limbs occurs because the medial portion of the lateral corticospinal tract, consisting of axons involved in the upper extremity function, is injured more than the lateral portion of the tract, which is mainly involved in the function of the legs [3]. Most frequently, the upper motor deficits have bilateral symmetry. Dickman et al. reported in a series of 14 patients that six presented with monoparesis [5,6].

Hopkins et al. demonstrated that 78.4% of the cases reported are traumatic with age being an important factor for recovery as patients over 60 years of age had worse outcomes compared to those younger than 60 [7]. Further, Dickman et al. suggested that surgical stabilization is more suitable in severe, unstable fractures with ligamentous injury. Such injuries are often associated with heavy trauma, and early rehabilitation may prove most beneficial for the patient [6].

Given the unstable nature of the patient’s injury and refusal to accept halo traction, an open reduction and fixation of the odontoid were performed. Although the patient’s neurological status in the immediate postoperative period was unchanged, there was a substantial return of strength in the upper extremities and overall improvement at the two-month follow-up. This unusual presentation illustrates yet another mechanism by which one may acquire this syndrome. It also underscores the importance of a thorough diagnostic evaluation and a broadened differential diagnosis when dealing with cervical spine trauma.

## Conclusions

Cruciate paralysis is a relatively rare although well-defined neurological condition which results from injury at pyramid decussation. In this case, the presence of a posterior bony spike of the fractured dens is responsible for the development of cruciate paralysis. Early diagnosis and adequate treatment can have successful results.
